# Clinical Intensity Modulated Proton Therapy for Hodgkin Lymphoma: Which Patients Benefit the Most?

**DOI:** 10.1016/j.prro.2019.01.006

**Published:** 2019-05

**Authors:** Georgios Ntentas, Katerina Dedeckova, Michal Andrlik, Marianne C. Aznar, Ben George, Jiří Kubeš, Sarah C. Darby, David J. Cutter

**Affiliations:** a Nuffield Department of Population Health, University of Oxford, Oxford, United Kingdom; b Proton Therapy Center Czech s.r.o., Prague, Czech Republic; c Manchester Cancer Research Centre, Division of Cancer Sciences, School of Medical Sciences, Faculty of Biology, Medicine and Health, The University of Manchester, Manchester, United Kingdom; d CRUK/MRC Oxford Institute for Radiation Oncology, Department of Oncology, University of Oxford, Oxford, United Kingdom; e Oxford Cancer Centre, Oxford University Hospitals NHS Foundation Trust, Oxford, United Kingdom

## Abstract

**Purpose:**

Radiation therapy (RT) improves control of Hodgkin lymphoma (HL), but patients who undergo RT are at risk for late effects, including cardiovascular disease and second cancers, because of radiation doses to organs at risk (OARs). Proton therapy (PT) can reduce OAR doses compared with conventional photon RT. However, access to PT is currently limited, so referrals must be appropriately selective. We aimed to identify subgroups of patients with HL who could benefit the most dosimetrically from RT with PT based on the prechemotherapy disease characteristics.

**Methods and materials:**

Normal tissue radiation doses were calculated for 21 patients with HL who were treated with deep-inspiration breath-hold pencil-beam scanning (PBS) PT and compared with doses from 3-dimensional conformal (3D-CRT) and partial arc volumetric modulated (PartArc) photon RT. Prechemotherapy disease characteristics associated with significant dosimetric benefits from PBS compared with photon RT were identified.

**Results:**

Treatment with PBS was well tolerated and provided with good local control. PBS provided dosimetric advantages for patients whose clinical treatment volume extended below the seventh thoracic level and for female patients with axillary disease. In addition, an increasing dosimetric benefit for some OARs was observed for increasing target volume. PBS significantly reduced the mean dose to the heart, breast, lungs, spinal cord, and esophagus. Dose homogeneity and conformity within the target volume were also superior with PBS, but some high-dose measures and hot spots were increased with PBS compared with partial arc volumetric modulated photon RT.

**Conclusions:**

PBS gives good target coverage and local control while providing reductions in radiation dose to OARs for individuals who receive RT for HL compared with advanced photon RT. Our findings highlight groups of patients who would be expected to gain more dosimetric benefit from PBS. These findings facilitate the selection of patients who should be considered a priority for PT.

## Introduction

Radiation doses to organs at risk (OARs) have been associated with an increased incidence of late effects in survivors of Hodgkin lymphoma (HL) who received radiation therapy (RT) in the past.^1^, ^2^, ^3^, ^4^, ^5^, ^6^, ^7^, ^8^, ^9^, ^10^ This means that clinicians are often willing to omit RT and accept the increased relapse rates and need for salvage therapy that occurs when chemotherapy is used alone.^11^ However, there have been technological advancements in RT, and incidental radiation to OARs from modern RT is often much lower than in the past.

Modern photon RT techniques, such as intensity modulated RT or advanced partial arc volumetric modulated RT (PartArc) have achieved substantial dose reductions to OARs.^12^, ^13^, ^14^, ^15^ An additional approach is proton therapy (PT), where steep dose gradients allow for further reductions in incidental radiation.^11^ In both photon RT and PT, the delivery of treatment in deep-inspiration breath-hold (DIBH) can decrease the dose to OARs even further.^12^, ^16^

A recent review by the Particle Therapy Cooperative Group Lymphoma Subcommittee of the use of PT for the treatment of HL summarized the results from 14 planning studies.^11^ This review found that PT reduced the dose to most OARs compared with conventional RT, such as 3-dimensional conformal (3D-CRT) or even advanced RT, such as intensity modulated RT and volumetric modulated arc therapy.

Access to PT is currently limited and more costly than conventional RT. Hence, even in developed countries, referrals must be appropriately selective for patients in whom dose escalation and OAR sparing is crucial. One method to determine which patients should be treated with PT is a model-based approach, for example, as adopted by the Health Council of the Netherlands.^17^ Using this method, both photon and proton treatment plans are produced for a patient who is considered for PT. Doses to the OAR from both plans are then integrated into normal tissue complication probability models to estimate and compare the risk of side-effects. Subsequently, patients who are more likely to benefit in terms of normal tissue complication probability reductions are chosen for PT. This is a systematic approach, but could be time consuming and expensive, especially if applied to all RT patients. To avoid these disadvantages, enabling some decisions without the need to produce RT treatment plans would be preferable.

### Aims of this study

In this study, we performed a dosimetric analysis using clinical data to identify subgroups of patients with HL who are likely to derive significant dosimetric benefits from PT. This would facilitate selection while reducing the number of patients who require detailed planning assessments, as is necessary with the Dutch methodology. We aimed to identify these patients based on pre-chemotherapy characteristics and before a treatment plan is produced. If the number of patients who need a detailed treatment planning assessment could be reduced, the selection process in a busy health care environment with scarce resources would be easier.

## Materials and Methods

### Patients

Between April 2015 and October 2016, 21 patients with HL (16 women and 5 men) were treated with involved site pencil-beam scanning (PBS) at 30 Gy (relative biological effectiveness of 1.1) in 15 fractions. All patients were irradiated in the supine position, with the arms alongside the body, and fixation via a 5-point head-and-neck mask with perforation for the accessories needed for DIBH (Fig. E1; available online at https://doi.org/10.1016/j.prro.2019.01.006). The planning computed tomography (CT) was acquired using the GE Optima CT 580W RT and in DIBH using the SDX system (DyńR-SDX, version 2.06). The study was approved by the institutional review board of the Proton Therapy Center Czech s.r.o.

### Contouring

The International Lymphoma Radiation Oncology Group (ILROG) recommendations^18^ were used for gross tumor volume and clinical target volume (CTV) definition. The heart and cardiac substructures were contoured by a radiation oncologist (D. Cutter) in accordance with the heart atlas by Feng et al.^19^

### Treatment planning

To make sure that an optimal plan was used for all treatment modalities, the clinical PBS treatment plans were created and delivered by medical physicists and radiation oncologists from the Proton Therapy Center Czech s.r.o. with clinical experience in PBS. The 3D-CRT and PartArc photon plans were created by medical physicists and radiation oncologists from the University of Oxford and the Oxford University hospitals NHS Foundation Trust with clinical experience in photon RT. Clinically realistic margins were used to create the planning target volumes (PTVs) for both photon and proton plans. A detailed description of the treatment planning methodologies is provided in the Appendix (available online at https://doi.org/10.1016/j.prro.2019.01.006).

### Dosimetric analysis

Clinically relevant dose measures were evaluated using dose volume histograms for both PTVs and OARs. The mean dose (D_mean_), dose received by the hottest 2% of the volume (D_2%_), maximum dose (D_max_), conformity index (CI), and homogeneity index (HI) were used to evaluate the PTV coverage. The conformity index was defined per the Radiation Therapy Oncology Group guidelines^20^ as V_95%_/PTV_volume,_ where V_95%_ was the body volume that received at least 95% of the prescribed dose. The HI was defined as (D_2%_-D_98%_)/(prescribed dose).

The mean dose to the normal tissue (NTD), which was defined as the whole body minus the PTV, and the integral dose to the normal tissue ((NTD) × (Body-PTV) volume) were also calculated. In terms of OAR dose measurements, D_mean,_ D_2%,_ D_max,_ and volumetric parameters such as V_5,_ V_10,_ V_20_, and V_30_ (ie, % volume of OAR that received 5 Gy, 10 Gy, 20 Gy, and 30 Gy, respectively) were extracted for the heart, lungs, breast, esophagus, larynx, thyroid, spinal cord, carotid arteries, and 12 cardiac substructures.

### Analysis of patient subgroups

To identify the subgroups of patients with HL for whom PBS would be a superior treatment, the techniques were compared for patients with different disease volumes and anatomies. The relationship between mean heart dose (MHD) and inferior mediastinal extent of the CTV was examined, and the same was done for mean lung dose (MLD). Specifically, patients were divided into 2 subgroups: those where the inferior border of the CTV was equal to or superior to the seventh thoracic level (T7) and those where this was inferior. T7 was selected as an easily identifiable landmark on diagnostic CT imaging with variable image quality. For each of these subgroups, a paired *t* test was used to compare the mean doses to each OAR for PartArc versus PBS and 3D-CRT versus PBS.

The mean breast dose (MBD) was assessed separately for patients with and without axillary involvement. For each of these subgroups, a paired *t* test was used to compare the mean doses to each OAR for PartArc versus PBS and 3D-CRT versus PBS.

The variation in the dosimetric benefit of PBS with increasing PTV was also examined. The mean NTD, MHD, MLD, and MBD were plotted against the PTV for all 3 treatments. PTV values were centered (ie, mean was subtracted). A linear regression was performed of each dose metric on centered PTV, and the significance of the slopes of the fitted regression lines were assessed. The statistical package used was STATA, version 14.2 (StataCorp, College Station, TX).

## Results

### Clinical outcomes and patient characteristics

The median follow-up time was 24 months (range, 13-38 months) with no reported recurrence or disease progression. Treatment was well tolerated by all patients, and no severe toxicities were reported. Minor acute toxicities were reported for some patients, including grade 1 dysphagia,^16^ grade 1 radiodermatitis,^6^ grade 2 leukopenia,^4^ grade 1 mucositis,^3^ and grade 1 anaemia.^1^ Other patient characteristics are shown in Table 1.

**Table 1:** Patient characteristics

	Number of patients	%
Sex		
Male	5	18
Female	16	82
Age at time of radiation therapy		
Median age: 31 years (range, 18-48 years)		
Risk factors		
Hypertension		
No	20	95
Yes	1	5
Hypercholesterolemia		
No	20	95
Yes	1	5
Smoking at time of Hodgkin lymphoma diagnosis		
No	16	82
Yes	5	18
Hodgkin lymphoma stage∗		
Early and intermediate (IIA/B)	18	86
Advanced (IIIB/IIISB)	3	14
Chemotherapy		
Median dose of anthracyclines 317.5 mg/m^2^, range (255-420)		
6 × escalated BEACOPP	4	19
2 × escalated BEACOPP + 2 × ABVD	15	71
4 × ABVD	2	10
Clinical target volume extension		
Below 7^th^ thoracic level	10	48
At & Above 7^th^ thoracic level	11	52
Axillary involvement		
Yes	9	43
No	12	57
Total number of patients	21	100

*Abbreviations:* ABVD = adriamycin, bleomycin, vinblastine, dacarbazine; BEACOPP = bleomycin, etoposide, doxorubicin, cyclophosphamide, vincristine, procarbazine and prednisolone.

∗ Lugano classification.^25^

### Dosimetric comparison

**Fig. 1:**
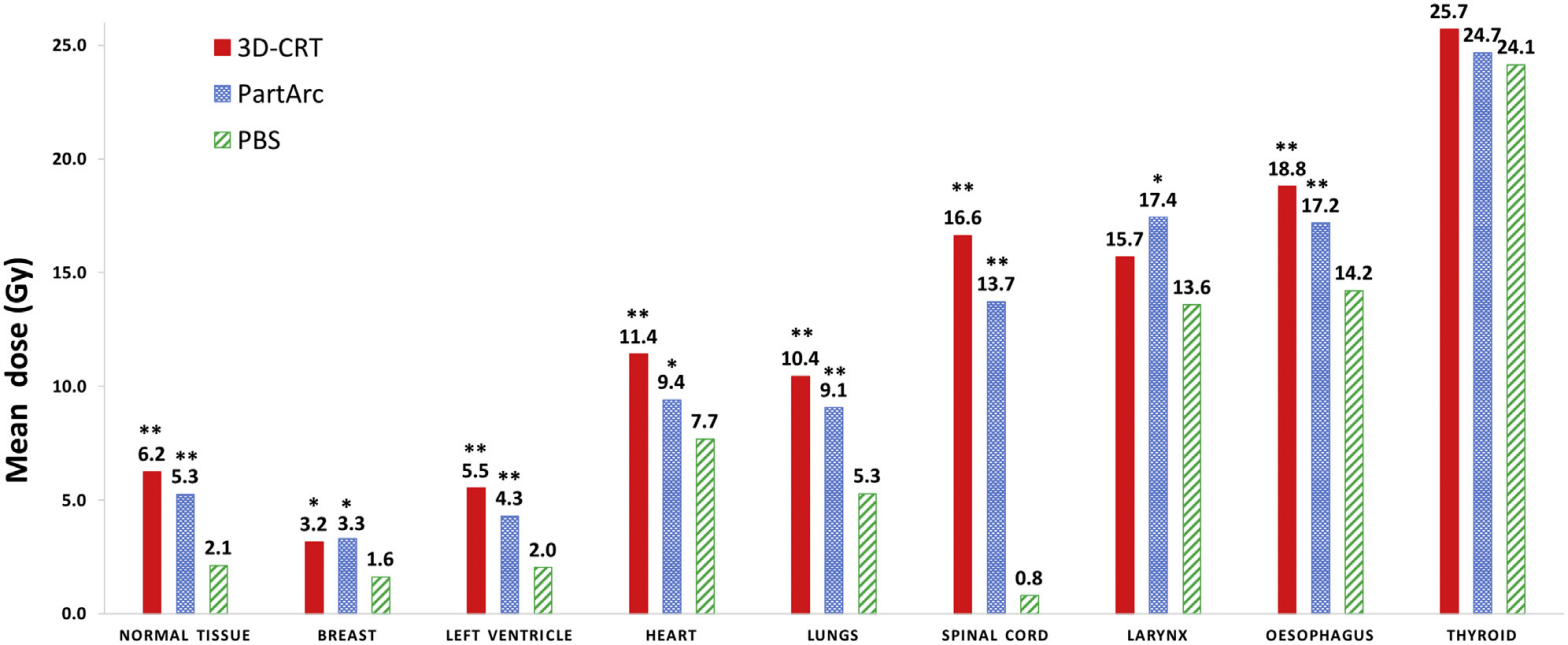
Dosimetric comparison: Mean dose to normal tissue and mean dose for various organs at risk for 3-dimensional conformal radiation therapy, partial arc volumetric modulated radiation therapy, and pencil beam scanning (PBS) proton therapy. **Significantly lower (*P* < .001) dose compared with PBS. *Significantly lower (*P* < .05) dose compared with PBS. *Abbreviations:* 3D-CRT = 3-dimensional conformal radiation therapy; PartArc = partial arc volumetric modulated; PBS = pencil beam scanning.

For all 21 patients, PTV coverage was acceptable for all RT techniques. However, PBS provided superior coverage and conformity for all patients compared with both photon techniques (Table E1; available online at https://doi.org/10.1016/j.prro.2019.01.006). PBS also provided advantages in terms of doses to OARs. The average MLD, MBD, and average of mean doses to the esophagus, larynx, and spinal cord were all significantly reduced with PBS (Fig. 1, Table 2). For these organs, the mean doses were lower for all patients with PBS than with either 3D-CRT or PartArc.

**Table 2:** Comparison of D_mean_, D_integral_, D_2%_, and maximum dose (D_max_) for organs at risk from 3D-CRT, PartArc, and PBS proton therapy

			Average values from 21 patients (Gy∗) *(range)*			Difference (Gy∗)† *P-value*	
Structure	Dose metric	Aims	3-dimensional conformal RT (A)	PartArc (B)	PBS‡ (C)	C minus A	C minus B
Normal tissue	D_mean_	-	6.2 *(3-10)*	5.3 *(3-8)*	2.1 *(1-4)*	−4.1 *P < .001*	−3.1 *P < .001*
	D_integral_	-	153.0 *(87-288)*	129.4 *(71-236)*	51.4 *(19-92)*	−100.9 *P < .001*	−76.1 *P < .001*
Breast	D_mean_	-	3.2 *(0-9)*	3.3 *(0-10)*	1.6 *(0-4)*	−1.5 *P = .004*	−1.7 *P = .007*
	D_2%_	-	24.4 *(2-34)*	20.9 *(5-34)*	19.2 *(1-30)*	−5.2 *P = .005*	−1.7 *P = .162*
Left Ventricle	D_mean_	-	5.5 *(0-23)*	4.3 *(0-18)*	2.0 *(0-10)*	−3.5 *P < .001*	−2.3 *P < .001*
	D_2%_	-	19.9 *(1-31)*	14.3 *(1-31)*	11.5 *(0-31)*	−8.5 *P < .001*	−2.9 *P = .001*
Heart	D_mean_	<10 Gy	11.4 *(1-28)*	9.4 *(1-23)*	7.7 *(1-15)*	−3.7 *P < .001*	−1.7 *P = .005*
	D_2%_	-	29.7 *(14-33)*	28.3 *(6-32)*	29.9 *(16-32)*	+0.2 *P = .662*	+1.5 *P = .009*
Lungs	D_mean_	<12 Gy	10.4 *(5-17)*	9.1 *(5-15)*	5.3 *(3-9)*	−5.2 *P < .001*	−3.8 *P < .001*
	D_2%_	-	31.8 *(30-35)*	30.0 *(29-31)*	30.9 *(29-32)*	−0.9 *P < .001*	+1.0 *P < .001*
Spinal Cord	D_mean_	-	16.6 *(9-27)*	13.7 *(7-24)*	0.8 *(0-2)*	−15.8 *P < .001*	−12.9 *P < .001*
	D_max_	<35 Gy	32.3 *(30-34)*	29.0 *(25-32)*	9.9 *(0-21)*	−22.4 *P < .001*	−19.1 *P < .001*
Larynx	D_mean_	-	15.7 *(0-31)*	17.4 *(0-29)*	13.6 *(0-30)*	−2.1 *P = .092*	−3.8 *P = .008*
	D_2%_	-	27.2 *(0-34)*	25.0 *(0-31)*	24.1 *(0-31)*	−3.1 *P < .001*	−0.9 *P = .174*
Oesophagus	D_mean_	-	18.8 *(14-28)*	17.2 *(12-23)*	14.2 *(3-19)*	−4.6 *P < .001*	−3.0 *P < .001*
	D_2%_	-	32.6 *(29-34)*	30.5 *(29-31)*	30.6 *(20-32)*	−1.9 *P < .001*	+0.1 *P = .787*
Thyroid	D_mean_	-	25.7 *(0-34)*	24.7 *(0-31)*	24.2 *(0-31)*	−1.6 *P = .482*	−0.5 *P = .820*
	D_2%_	-	30.0 *(0-35)*	28.4 *(0-33)*	28.1 *(0-32)*	−1.9 *P < .001*	−0.3 *P = .126*
Carotid arteries	D_mean_	-	27.9 *(13-33)*	26.4 *(12-30)*	27.3 *(13-31)*	−0.6 *P = .03*	+0.9 *P < .001*
	D_2%_	-	33.2 (32-35)	31.2 (31-32)	31.3 (31-32)	−1.8 *P < .001*	−0.1 *P = .14*

*Abbreviations:* 3D-CRT = 3-dimensional conformal RT; D_2%_ = dose received by the hottest 2% of the volume; D_integral_ = mean integral dose; D_mean_ = mean dose; PartArc = partial arc volumetric modulated; PBS = pencil beam scanning; RT = radiation therapy.

∗ Joule for D_integral_.

† Negative values indicate that PBS decreased the dose compared with the respective photon technique, and positive values indicate that PBS increased the dose compared with the respective photon technique.

‡ PBS in Gy (relative biological effectiveness = 1.1).

The NTD and integral dose were also significantly lower with PBS compared with both 3D-CRT and PartArc. The average MHD and mean left ventricular dose were also significantly reduced compared with both photon methods, but for 4 patients, the MHD received with PBS was higher than with PartArc. There was no significant difference in thyroid dose between the PBS and either photon RT plan. The mean dose to the carotids was significantly decreased with PBS compared with 3D-CRT, but significantly increased compared with PartArc.

PBS also increased D2% compared with PartArc for the lungs, heart, and esophagus, as well as some other high-dose volumetric parameters, including V30 (Table E2; available online at https://doi.org/10.1016/j.prro.2019.01.006). The dosimetric differences were also assessed for 12 cardiac substructures (Table E3; available online at https://doi.org/10.1016/j.prro.2019.01.006).

### Dosimetric benefits for patient subgroups

**Fig. 2:**
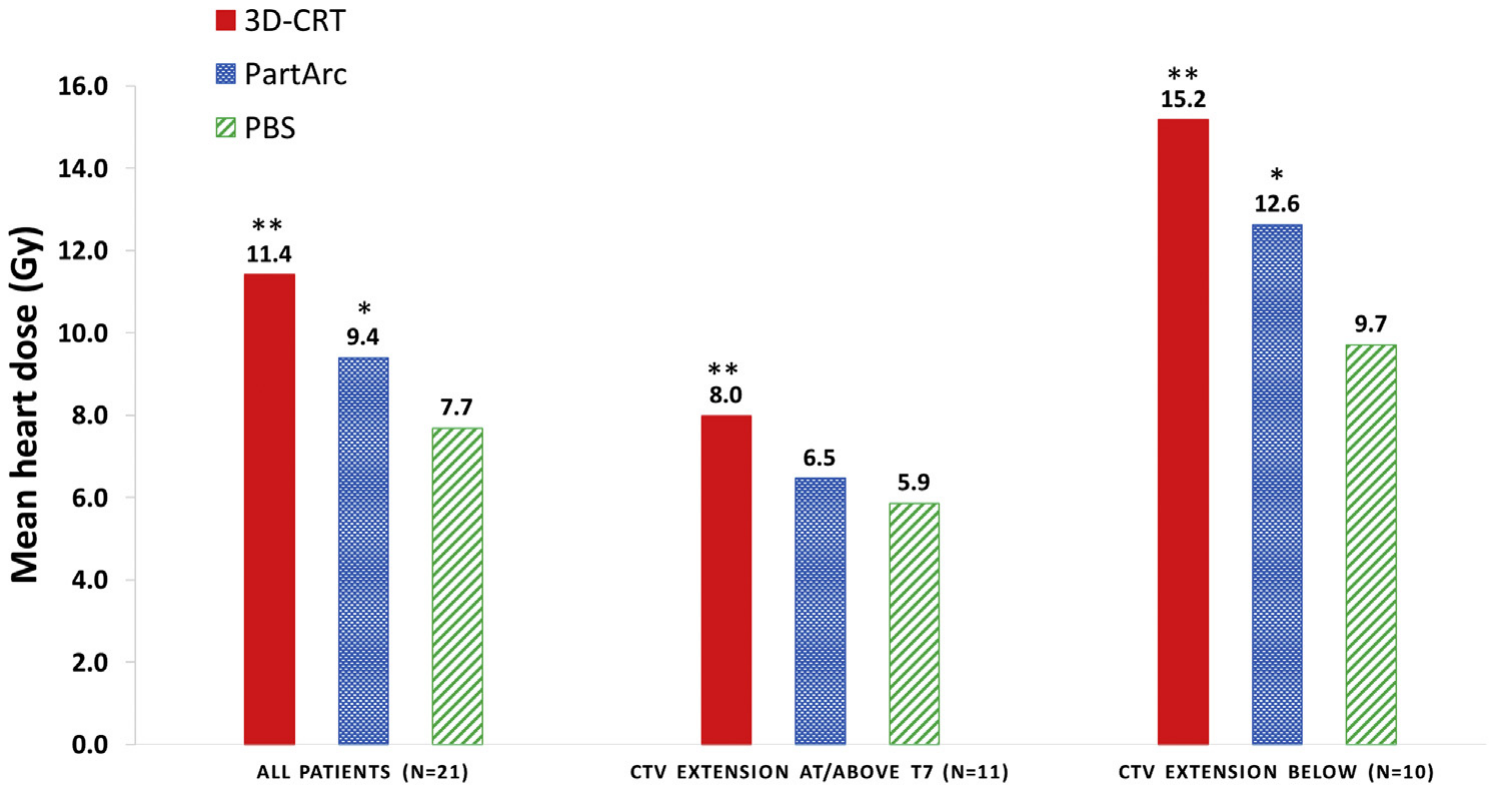
Mean heart dose for 3-dimensional conformal radiation therapy, partial arc volumetric modulated radiation therapy, and pencil beam scanning (PBS) proton therapy according to clinical target volume thoracic level. **Significantly lower (*P* < .001) dose compared with PBS. *Significantly lower (*P* < .05) dose compared with PBS. *Abbreviations:* 3D-CRT = 3-dimensional conformal radiation therapy; PartArc = partial arc volumetric modulated; PBS = pencil beam scanning.

Compared with 3D-CRT, PBS significantly reduced the MHD both overall (reduction of 3.7 Gy; *P* < .001) and for both subgroups of patients when considered separately according to CTV extension (reductions of 2.1 Gy; *P* < .001 for CTV at or above T7; and 5.4 Gy; *P* < .001 for CTV below T7; Fig. 2). Compared with PartArc, the reduction in MHD was significant overall (reduction of 1.7 Gy; *P* < .05) and for the patient group with a CTV extension below T7 (reduction of 2.9 Gy; *P* < .01), but not for the group with a CTV extension at or above T7 (reduction of 0.6 Gy; *P* = .22). PBS reduced MLD overall compared with both 3D-CRT and PartArc (reductions of 5.2 Gy; *P* < .001, and 3.8 Gy; *P* < .001, respectively), but no additional dosimetric benefit was observed when patients were considered separately according to CTV extension.

**Fig. 3:**
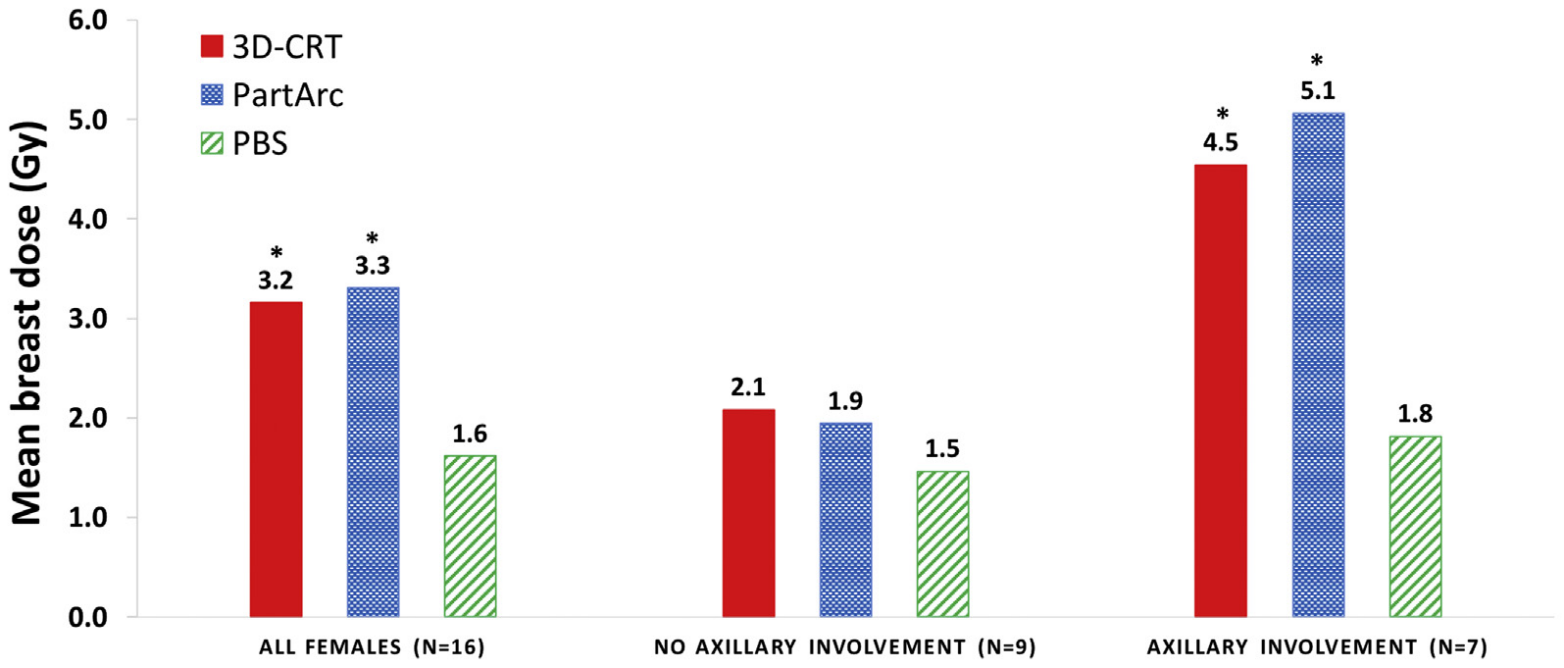
Mean breast dose for 3-dimensional conformal radiation therapy, partial arc volumetric modulated radiation therapy, and pencil beam scanning (PBS) proton therapy for all female patients and patients with and without axillary involvement. **Significantly lower (*P* < .001) dose compared with PBS. *Significantly lower (*P* < .05) dose compared with PBS. *Abbreviations:* 3D-CRT = 3-dimensional conformal radiation therapy; PartArc = partial arc volumetric modulated; PBS = pencil beam scanning.

For female patients with axillary disease, PBS reduced the MBD significantly compared with either of the photon RT methods (reductions of 2.7 Gy; *P* < .01 and 3.3 Gy; *P* < .01 compared with 3D-CRT and PartArc, respectively; Fig. 3). For patients with no axillary involvement, the dosimetric benefit of PBS versus either 3D-CRT or PartArc was not statistically significant (reductions of 0.6 Gy; *P* = .06 and 0.4 Gy; *P* = .09, respectively).

### Dosimetric benefits versus planning target volume

**Fig. 4:**
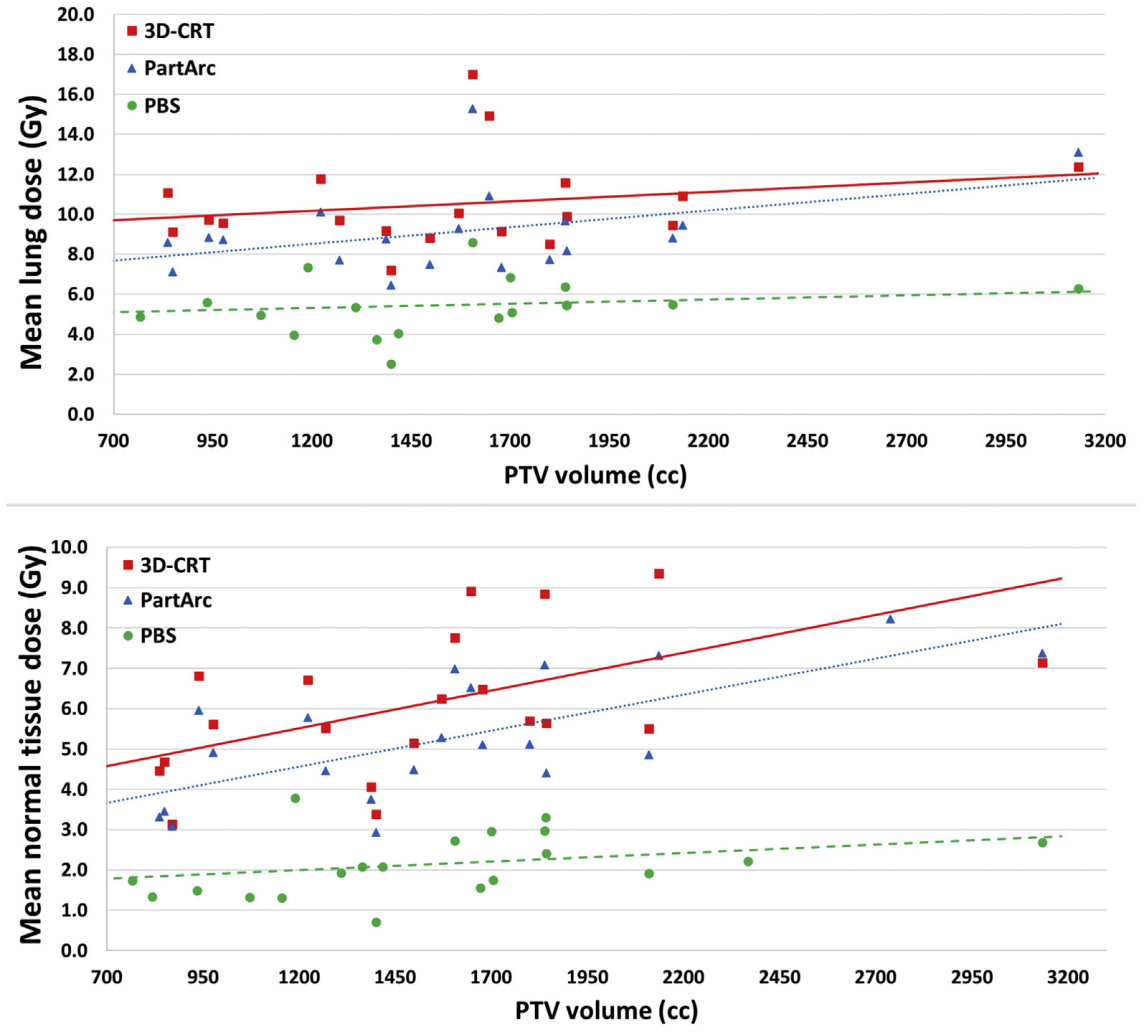
Relationship between mean lung dose (MLD) and planning target volume (PTV, top panel) and among mean normal tissue dose and PTV (bottom panel) for 3-dimensional conformal radiation therapy (RT), partial arc volumetric modulated RT, and pencil beam scanning (PBS) proton therapy. The fitted regression models for MLD describing the three radiation therapy methods were 3-dimensional conformal RT (y = 0.002x + 7.5; *P* = .05), partial arc volumetric modulated RT (y = 0.002x + 5.8; *P* = .01), and pencil beam scanning (y = 0.0004x + 4.6; *P* = .44), where y is the MLD and x is the PTV. The fitted regression models for mean normal tissue dose were 3-dimensional conformal RT (y = 0.002x + 3.3; *P* = .005), partial arc volumetric modulated RT (y = 0.002x + 2.4; *P* < .011), and PBS (y = 0.0004x + 1.5; *P* = .16), where y is the normal tissue dose, and x is the PTV volume. *P* values indicate the significance of the trend in the linear regression model. *Abbreviations:* 3D-CRT = 3-dimensional conformal radiation therapy; PartArc = partial arc volumetric modulated; PBS = pencil beam scanning; PTV = planning target volume.

For PBS, the MLD did not increase significantly with increasing PTV (p_trend_ = .44), but in contrast for both photon RT techniques, the MLD increased significantly with increasing PTV (p_trend_ = .05 for 3-dimensional conformal RT and p_trend_ = .01 for PartArc; Fig. 4 - top panel). The MLD increased by 0.5 Gy per 250 cc of PTV when using either photon RT technique.

The pattern for NTD was similar to that for MLD, and the NTD did not increase significantly with increasing PTV for PBS (p_trend_ = .16). On the other hand, for the photon RT techniques, the NTD increased significantly with increasing PTV (p_trend_ = .005 for 3D-CRT and p_trend_ < .001 for PartArc; Fig. 4 - bottom panel). Once again, there was an increase of 0.5 Gy of NTD per 250 cc of PTV when using either photon RT technique.

## Discussion

This is the only group of patients with HL treated with DIBH-PBS for which clinical follow-up and a comprehensive dosimetric analysis has been reported. In addition, this is the first study that used prechemotherapy disease characteristics to identify patients with HL who would be expected to derive the greatest dosimetric benefit from PT. Our results add evidence to the recent PT guidelines for adults with mediastinal lymphoma published by ILROG, which identify the need for evidence-based case selection for PT.^21^ The ILROG guidelines identified that patients with disease below the left main stem coronary artery (LMSCA) will benefit most from PBT. Our choice of T7 as a landmark not only correlates with the position of the LMSCA but is also more easily identifiable on diagnostic CT imaging when LMSCA is often not clearly seen owing to lack of contrast.

In terms of dosimetric benefits, PBS performed better for most dose metrics when compared with either photon RT technique. PBS significantly reduced the mean dose to most OAR and improved the dose homogeneity and conformity within the target volume. However, some high dose parameters and hot spots were increased with PBS compared with PartArc. To manage the effect of these high dose areas, planning optimization and motion management are important to ensure that organs or cardiac substructures are not included because the impact of hot spots within critical structures would be expected to increase risk. For example, inhomogeneity of dose within the heart and the dose to the coronary arteries were reported as important predictors of late cardiotoxicity in a recent study by Hahn et al.^22^

Our results show that, although the reduction in dose using PBS can be substantial, not all patients benefit equally from this scarce and expensive treatment modality. The hypothesis that patients with specific disease characteristics could benefit considerably from PBS was confirmed. The MHD was substantially reduced for patients with CTV that extended below T7, and MBD reduced for female patients with axillary disease. Therefore, these patients comprise a group who could be preferentially considered for PBS.

PBS was also found to provide an increasing dosimetric advantage for patients with more extensive disease and hence a larger PTV. A reduction of 0.5 Gy of MLD and NTD per 250 cc of increase in PTV was observed relative to both photon RT methods. Therefore, the larger the PTV, the greater the dosimetric benefit provided by PBS in terms of MLD and NTD. However, the same conclusion cannot be drawn for MHD because MHD can be affected substantially by the anatomical relationship of the disease to the heart. Therefore, the dosimetric benefit for the heart may not increase with increasing PTV as for the lungs and nontarget body. There were insufficient patients in our series to classify them into separate groups large enough to examine this effect.

### Strengths and limitations of the study

In this study, the most advanced methods of both treatment modalities were used, and maximum efforts were made to create optimal plans. Treatment plans were created by experts with experience in both proton and photon RT from experienced clinical departments. Clinically realistic margins were used to create the PTVs for both photon RT and PBS separately rather than using the same PTV. Patients with a variety of disease volumes and field arrangements (anterior-posterior only, anterior-posterior and posterior-anterior, posterior-anterior only) were included in the study to account for the wide ranges of disease burden and typical location of HL.

To our knowledge, no other study of patients with HL has identified potential indicators for referral for PT based on disease location or extent of involvement. Furthermore, this is the only DIBH-PBS study to our knowledge to report on the dose to a large number of cardiac substructures. Twelve cardiac substructures were contoured, and dosimetric information was provided for all. This is important because MHD is not the only dosimetric parameter that can be used to predict cardiac toxicity. The dose to substructures, such as the left ventricle,^3^ valves,^23^ and coronary arteries^2^ have been reported to be predictors for specific cardiac endpoints.

Even though this was the largest number of patients with HL treated with DIBH-PBS reported on in the literature, the patient number (n = 21) was still low. Studies including larger numbers of patients are needed to strengthen the conclusions on the dosimetric benefits of PBS, and particularly to identify patient groups that might benefit the most. In addition, patients in this study were specifically referred for PT and may not represent the full range of patients with HL in terms of disease distribution and treatment volume. Mediastinal and more extensive disease usually results in higher OAR doses, and the dosimetric benefits may be larger in these patients than in those with more limited disease who were not referred for PT.

Different margins were used for protons and photons when creating the PTV, but the optimal proton PTV margins are not known yet. Unless all current proton uncertainties are quantified and included in the margins, at least to the extent of current photon uncertainties, there will always be an inherent uncertainty in dosimetric comparisons between protons and photons. In addition, a decision was made to reduce the photon margins by 5.0 mm in the superior-inferior CTV borders because the plans were delivered in DIBH. Even though the use of DIBH is increasing in HL RT, the majority of centers worldwide still deliver in free breathing.

The additional dose deposited by neutrons was not reported in this study. However, studies have shown that neutron dose during PBS is measured as comparable, if not less than that received by high-energy photon RT.^24^ The inclusion of an additional neutron dose is unlikely to have had a material effect on the overall conclusions of this study.

## Conclusions

In this study, we showed that patients with HL with a CTV that extends below the seventh thoracic level, female patients with axillary disease, and patients who have more extensive disease and hence a larger PTV derive significant dosimetric benefit from PBS treatments. To our knowledge, this is the first study to identify subgroups of patients with HL for whom DIBH-PBS could provide additional dosimetric benefits compared with DIBH-photon RT. These findings may facilitate the efficient selection of patients who should be considered a priority for PBS in resource-limited health care environments.
