# Supplementary material for: Clinical Intensity Modulated Proton Therapy for Hodgkin Lymphoma: Which Patients Benefit the Most?
Source: Pract Radiat Oncol. 2019 May;9(3):179–87. doi: 10.1016/j.prro.2019.01.006 (PMC6493042; doi:10.1016/j.prro.2019.01.006)
Supplement: Supplementary Material [file mmc1.docx]

**Appendix**

**E1. Treatment planning**

The same PTV coverage aims and organs at risk (OAR) dose restrictions were used for all RT techniques (Tables E1 and E2) There were no specific dose constraints placed on any cardiac substructures.

# E1.1 PBS planning

For the proton therapy plans the clinical target volume (CTV) was divided into two parts, a neck and a mediastinal section. A margin of 5 mm was added to the mediastinal section of the CTV to account for uncertainties for internal organ motion in the thoracic area such as the lungs and heart. The resulting structure was referred to as the proton internal target volume (Proton-ITV). An additional uniform margin of 8 mm was added to the Proton-ITV as well as to the CTV in the neck section to create the proton planning target volume (Proton-PTV), i.e. the target volume for PBS. The proton plans were created using XiO TPS (Version V4.80.00.7, Elekta CMS, St. Louis, MO) and were delivered by an IBA Proteus®PLUS proton therapy system. The Pencil Beam Algorithm was used for the dose calculation.

The proton plans were generated using the following approach, a single anterior field with gantry angle 0° was used for upper-mediastinal involvement, a three-field solution was used when neck and axillary nodes were involved (0°, 60° and 300°). A fourth field (180°) was introduced for cases with additional lower mediastinal involvement. Those gantry angles varied slightly depending on the patient anatomy and the target volume location. The PBS treatment times were usually between 15 and 25 minutes per patient, depending on treatment specifications. The beam-on time varied between one and eight minutes according to the number of fields used. The number of breath-holds varied between 2 and 15.

**E1.2 Photon RT planning**

For the photon radiotherapy plans, a Photon-PTV was created using different margins than those used for protons. This is due to the different physical properties between protons and photons. For protons and other charged particles, the effect of set-up errors on the position of isodoses is considerably less in the direction of the incident beam than it is laterally. A uniform 10 mm margin was added to the CTV, also following the ILROG guidelines. However, as advised by the guidelines, a larger margin was not added in the superior-inferior direction as the treatment would be delivered in DIBH.

The Eclipse TPS (Version 13.0.28, Varian Medical System, Palo Alto, CA) was used to produce the PartArc and 3D-CRT treatment plans and those were approved by a radiation oncologist as clinically acceptable. The Anisotropic Analytical Algorithm version 13.0.26 (AAA_13026) was used for both photon techniques for dose calculation and the plans were normalised on the median for the PartArc and at 95% of the dose covering 95% of the photon-PTV for the 3D-CRT respectively in order to reflect clinical practice.

**E1.2.1 PartArc**

For every patient, clinically deliverable photon radiotherapy plans (PartArc and 3D-CRT) were produced in a different institution by experienced photon therapy planners and approved by a clinical oncologist.

The PartArc approach consisted of one anterior and one posterior arc of 60^o^ to 70^o^ depending on the photon-PTV size and one non-coplanar arc of 60^o^ with gantry rotation from 330^o^ to 30^o^ and couch rotation at 90^o^. The beam energy was 6 megavolts (MV). Similar approaches have been previously described (14), (15) When the photon-PTV extended to the neck area, in order to maintain good coverage and dose conformality with no sacrifice in normal tissue dose or allowing for hot spots, an additional full arc of 360^o^ (gantry rotation from 181 ^o^ to 179 ^o^) that only covered the neck and upper supraclavicular area was added (for 13 out of 21 patients) (Fig. E1).

#####
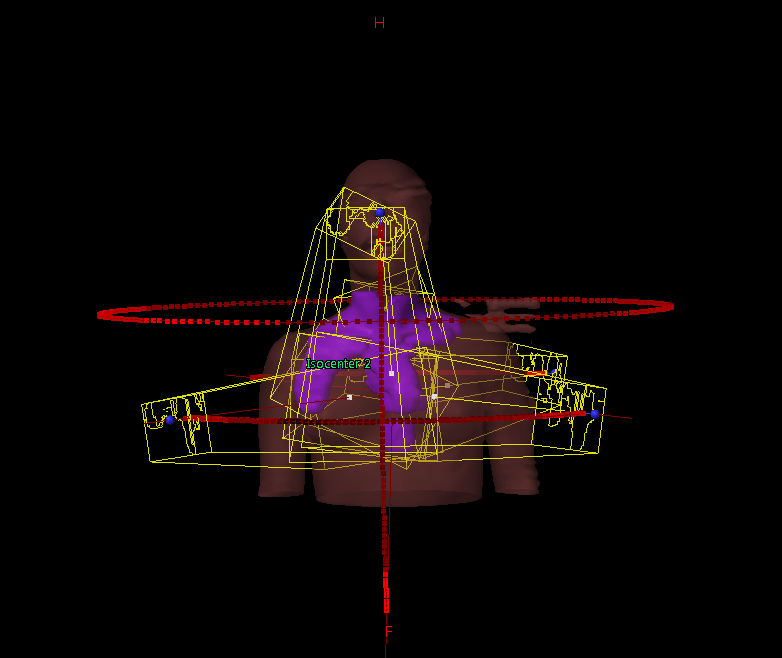

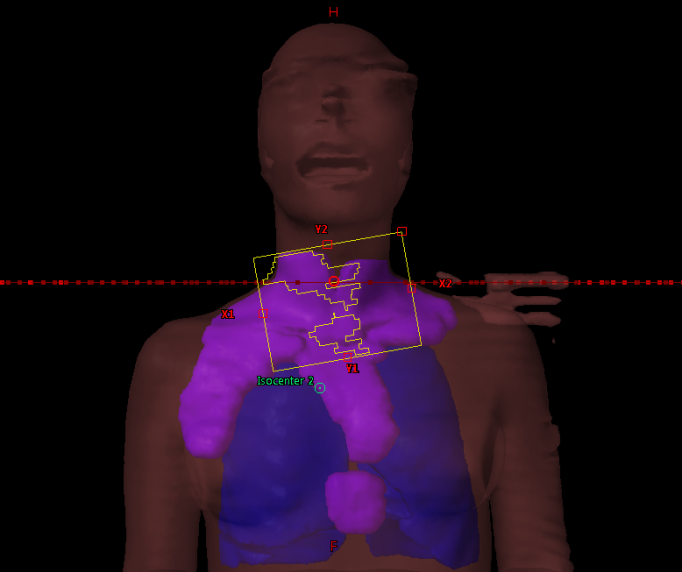


Fig. E1 Arc arrangement in PartArc method with additional full arc around the neck and supraclavicular area. Left panel: anterior view of the patient, the standard PartArc arrangement and the added full arc can be seen in red outline around the patient contour. Right panel: Beam’s eye view of the additional full arc. It can be clearly seen that the field only includes parts of the photon-PTV (purple) of the supraclavicular and neck area and avoids OAR such as lungs (blue) to reduce low dose bath.

# E1.2.2 3D-CRT

The field-in-field 3D-CRT technique included two main opposed fields, one anterior at 0^o^ and one posterior at 180^o^, as well as smaller sub-fields of the same angles. The beam energy was 6 MV. For patients with a large AP-PA separation at the level of the mediastinum, the beam energy of the sub-fields was adjusted to 10 MV when this improved PTV coverage.

#####
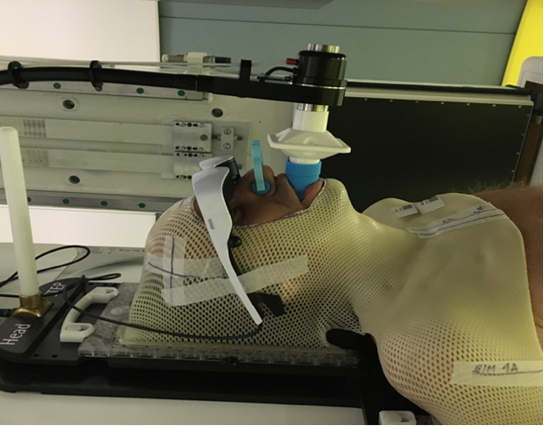


Fig. E2 Five-point head and neck mask used for body fixation to reduce patient motion during treatment. A deep inspiration breath-hold SDX® system was used to monitor patients breathing during treatment. (Image reproduced with permission from the Proton Therapy Center Czech s.r.o.)

Table E1 Comparison of dosimetric parameters between proton and photon therapy for planning target volume, (PBS versus 3D-CRT and PartArc).

| **Dose metric** | **Aims** | **3D-CRT** | **PartArc** | **PBS^‡^** |
| --- | --- | --- | --- | --- |
| **D_mean_ (Gy ± SD)** | *30.0* | *31.3 ±0.6* | *29.9 ±0.3* | *30.8 ±0.3* |
| **D_2%_ (Gy ± SD)** | *-* | *33.8 ±0.9* | *31.6 ±0.4* | *31.4 ±0.3* |
| **D_max_ (Gy ± SD)** | *< 33.0* | *34.7 ±0.9* | *33.5 ±0.8* | *32.5 ±0.6* |
| **V95% (%)** | *> 95.0* | *94.9 ±1.1* | *95.9 ±2.6* | *98.9 ±1.0* |
| **Conformity Index (CIx)** | *1.0* | *2.94* | *1.38* | *1.30* |
| **Homogeneity Index (HI)** | *0.0* | *0.21* | *0.12* | *0.08* |
| **PTV Volume (cc ± SD)** | *-* | *1519.6 ±579.3* | *1519.6 ±579.3* | *1587.3 ±600.0* |

^‡^Pencil beam scanning in Gy (RBE = 1.1).

SD: Standard deviation.

PTV: Planning target volume.

Table E2 Comparison of volumetric parameters between proton and photon therapy for the main organs at risk following HL radiotherapy, (PBS versus 3D-CRT and PartArc).

|  |  |  | **Average values from**  **21 patients** | | | **Difference**^¶^ | |
| --- | --- | --- | --- | --- | --- | --- | --- |
| **Structure** | **Dose metric** | **Aims** | **3D-CRT**  **(A)** | **PartArc (B)** | **PBS**  **(C)** | **C minus A** | **C minus B** |
| **Heart** | **V5 (%)** | *-* | *42* | *42* | *33* | ***-8**** | ***-9**** |
|  | **V10 (%)** | *-* | *39* | *34* | *28* | ***-11**** | ***-6**** |
|  | **V20 (%)** | *-* | *33* | *24* | *20* | ***-13**** | *-4****^†^*** |
|  | **V30 (%)** | *<10* | *10* | *4* | *11* | *1****^†^*** | ***7***** |
| **Lungs** | **V5 (%)** | *<50* | *42* | *45* | *24* | ***-18***** | ***-21***** |
|  | **V10 (%)** | *-* | *34* | *33* | *20* | ***-14***** | ***-14***** |
|  | **V20 (%)** | *<25* | *27* | *21* | *14* | ***-13***** | ***-7***** |
|  | **V30 (%)** | *-* | *13* | *2* | *6* | ***-8***** | ***4***** |
| **Breast** | **V4 (%)** | *<20* | *12* | *19* | *10* | *-2****^†^*** | ***-9**** |
|  | **V5 (%)** | *<20* | *11* | *17* | *9* | *-2****^†^*** | ***-8**** |
|  | **V10 (%)** | *<10* | *9* | *11* | *7* | *-2****^†^*** | *-4****^†^*** |
|  | **V20 (%)** | *-* | *7* | *5* | *3* | *-3****^†^*** | *-2****^†^*** |
|  | **V30 (%)** | *-* | *4* | *1* | *0* | ***-4**** | *-1****^†^*** |

** indicates significantly lower (p<0.001) dose with PBS.

* indicates significantly lower dose (p<0.05) with PBS.

**^†^** indicates not significantly different

^¶^Negative values indicate that PBS decreased the dose compared to the respective photon technique, while positive values indicate that PBS increased the dose compared to the respective photon technique.

‡Pencil beam scanning in Gy (RBE = 1.1).

Table E3 Comparison of mean dose (Dmean), maximum dose (Dmax) and the dose received by the hottest 2% of the volume (D2%) for 12 cardiac substructures (PBS versus 3D-CRT and PartArc).

|  |  | **Average dose (Gy)** | | | **Difference**^¶^ | |
| --- | --- | --- | --- | --- | --- | --- |
| **Structure** | **Dose metric** | **3D-CRT (A)** | **PartArc (B)** | **PBS*^‡^***  **(C)** | **C minus A** | **C minus B** |
| **Aortic Valve** | **D_mean_** | *24.0* | *20.0* | *15.7* | ***-8.4***** | ***-4.3***** |
|  | **D_2%_** | *26.5* | *25.0* | *25.3* | *-1.2****^†^*** | *0.3****^†^*** |
|  | **D_max_** | *26.9* | *25.9* | *26.7* | *-0.1****^†^*** | *0.8****^†^*** |
| **Pulmonary Valve** | **D_mean_** | *25.8* | *23.8* | *25.1* | *-0.6****^†^*** | *1.3****^†^*** |
|  | **D_2%_** | *27.9* | *27.0* | *28.7* | *0.8****^†^*** | ***1.7**** |
|  | **D_max_** | *28.3* | *27.6* | *29.0* | *0.7****^†^*** | *1.4****^†^*** |
| **Mitral Valve** | **D_mean_** | *11.6* | *8.1* | *2.1* | ***-9.6***** | ***-6.1***** |
|  | **D_2%_** | *15.4* | *11.4* | *4.2* | ***-11.2***** | ***-7.3***** |
|  | **D_max_** | *16.4* | *12.3* | *5.0* | ***-11.4***** | ***-7.3***** |
| **Tricuspid Valve** | **D_mean_** | *7.4* | *5.9* | *3.2* | ***-4.2**** | ***-2.7**** |
|  | **D_2%_** | *10.7* | *8.9* | *6.1* | ***-4.6**** | ***-2.8**** |
|  | **D_max_** | *11.4* | *9.6* | *7.3* | ***-4.1**** | *-2.3****^†^*** |
| **LAD** | **D_mean_** | *10.4* | *9.0* | *9.1* | *-1.3****^†^*** | *0.1****^†^*** |
|  | **D_2%_** | *23.1* | *18.9* | *19.0* | ***-4.1**** | *0.1****^†^*** |
|  | **D_max_** | *25.5* | *20.5* | *20.2* | *-5.4****^†^*** | *-0.3****^†^*** |
| **L C Coronary Art.** | **D_mean_** | *25.8* | *21.7* | *20.3* | ***-5.6**** | *-1.5****^†^*** |
|  | **D_2%_** | *27.6* | *24.7* | *23.1* | ***-4.5**** | *-1.6****^†^*** |
|  | **D_max_** | *27.9* | *25.4* | *23.9* | ***-4.1**** | *-1.5****^†^*** |
| **R Coronary Art.** | **D_mean_** | *16.1* | *13.7* | *15.3* | *-0.8****^†^*** | *1.6****^†^*** |
|  | **D_2%_** | *25.7* | *24.9* | *25.9* | *0.3****^†^*** | *1.1****^†^*** |
|  | **D_max_** | *26.1* | *25.5* | *26.5* | *0.5****^†^*** | *1.0****^†^*** |
| **L Atrium** | **D_mean_** | *18.2* | *13.4* | *6.0* | ***-12.2**** | ***-7.4**** |
|  | **D_2%_** | *25.3* | *23.3* | *17.8* | ***-7.5**** | ***-5.5**** |
|  | **D_max_** | *26.6* | *24.8* | *21.9* | ***-4.7**** | *-2.9****^†^*** |
| **R Atrium** | **D_mean_** | *14.2* | *11.2* | *10.0* | ***-4.2**** | *-1.2****^†^*** |
|  | **D_2%_** | *26.6* | *26.6* | *27.1* | ***0.5**** | *0.5****^†^*** |
|  | **D_max_** | *28.5* | *29.1* | *29.3* | ***0.7**** | *0.2****^†^*** |
| **R Ventricle** | **D_mean_** | *8.5* | *6.8* | *6.7* | ***-1.8**** | *-0.1****^†^*** |
|  | **D_2%_** | *25.3* | *23.8* | *26.0* | *0.7****^†^*** | ***2.2**** |
|  | **D_max_** | *26.7* | *26.8* | *27.6* | ***0.9**** | *0.8****^†^*** |
| **L Circumflex** | **D_mean_** | *10.4* | *8.5* | *5.7* | ***-4.7***** | ***-2.8***** |
|  | **D_2%_** | *23.7* | *18.2* | *17.0* | ***-6.7**** | *-1.2****^†^*** |
|  | **D_max_** | *24.5* | *19.6* | *18.4* | ***-6.1**** | *-1.1****^†^*** |

** indicates significantly lower (p<0.001) dose with PBS.

* indicates significantly lower dose (p<0.05) with PBS.

**^†^** indicates not significantly different

^¶^Negative values indicate that PBS decreased the dose compared to the respective photon technique, while positive values indicate that PBS increased the dose compared to the respective photon technique.

‡Pencil beam scanning in Gy (RBE = 1.1).

L C Coronary Art: Left circumflex coronary artery

LAD: Left Anterior Descending (including left main stem).
